# Development of the pictorial values recognition test: assessing middle school students’ value perceptions

**DOI:** 10.3389/fpsyg.2025.1736681

**Published:** 2026-01-09

**Authors:** Derya Kayıran

**Affiliations:** Department of Child Development, Kahramanmaras Sütçü Imam University, Kahramanmaras, Türkiye

**Keywords:** middle school students, picture based values questionnaire, value recognition, values education, visual based values assessment, pictorial values scale for students

## Abstract

**Introduction:**

The purpose of this study was to develop and validate the *Pictorial Values Recognition Test (PVRT)*, a visual-based measurement tool designed to assess middle school students’ ability to recognize 12 values. Values education is a critical component of moral and social development; however, conventional assessment methods often fail to capture students’ perceptions of values in contextual or visual formats. This study addresses this gap by introducing a reliable and contextually grounded assessment instrument.

**Method:**

A quantitative survey design was employed. The study group consisted of 336 middle school students enrolled in public schools across different regions of Türkiye during the 2025–2026 academic year. The PVRT comprised 12 items, each depicting an illustration representing a distinct value such as justice, honesty, respect, compassion, and responsibility. Students identified the value associated with each picture. Test scores ranged from 0 to 12, categorized as low (0–4), moderate (5–8), and high (9–12) levels of value recognition.

**Results:**

The findings showed that 64.3% of students demonstrated a moderate level, 32.7% a high level, and 3% a low level of value recognition. The KR-20 reliability coefficient (0.87), along with acceptable item difficulty (0.58–0.82) and discrimination indices (0.38–0.55), confirmed the internal consistency and validity of the test.

**Discussion and conclusion:**

The results highlight that visual-based assessment tools can effectively facilitate students’ understanding of abstract moral and social concepts. The PVRT offers educators a valid and reliable instrument for assessing value recognition and emphasizes the pedagogical value of incorporating visual materials into values education to enhance students’ engagement and comprehension.

## Introduction and literature review

Values are normative frameworks that guide beliefs, choices, and social interactions. In school settings, values are not merely cognitive content; they are entwined with social cohesion, belonging, and civic dispositions. Recent work demonstrates that shared values among teachers and students covary with school climate, prosocial behavior, and indicators of wellbeing (Luo et al., 2023; Oeschger et al., 2024; Wang et al., 2022). As the relational features of school climate (trust, respect, inclusiveness) intersect with social–emotional competencies, values-based initiatives can strengthen empathy, cooperation, and citizenship tendencies (Kim and Park, 2024; Silke, 2024).

Early adolescence (ages 11–14) represents a developmentally sensitive window for the recognition and internalization of values. During this period, social cognition and executive control expand while peer influence intensifies; together, these forces rapidly shape value orientations and classroom behavior (Cheng, 2024). Longitudinal and comparative studies indicate that teachers’ value-related educational goals and the value-related school climate co-evolve, reinforcing what is modeled, rewarded, and normed in daily practice (Oeschger et al., 2024). These findings amplify the need for instruments that can validly assess values recognition at the middle-school level, in ways that are sensitive to context and development.

Two recent trends characterize measurement in this area. First, many studies still anchor to Schwartz’s theory of basic human values while adapting tasks to child and adolescent populations (e.g., recognition or prioritization of specific value content). Second, scale development now more consistently follows contemporary validity and reporting standards gathering evidence from test content and response processes to internal structure, relations to other variables, and the intended consequences of testing (American Educational Research Association, American Psychological Association, and National Council on Measurement in Education, 2014; Brown and Zhao, 2023; Sireci, 2023). For new instruments, this implies reporting clear construct definitions, cognitive interviews for response-process evidence, exploratory/confirmatory factor analyses (EFA/CFA), internal consistency and stability estimates, measurement invariance across salient groups (e.g., sex, grade), and theoretically expected correlations with external criteria (e.g., school climate, prosocial behavior).

A methodological gap remains, however, for middle-school assessments that move beyond decontextualized Likert items and evaluate values recognition through situated, pictorial cues. Visual and pictorial tasks can make abstract values concrete, capture attention, and elicit faster, context-sensitive judgments of “which behavior exemplifies which value” in recognizable school scenarios. In educational research, visually supported assessments have been designed and validated for communicative and cognitive skills, demonstrating that digital/visual tasks can be psychometrically sound while improving engagement (Lo and Huang, 2024; Simon et al., 2022). Translating this approach to values recognition is promising precisely because many school-based values (e.g., fairness, responsibility, respect, helping) are enacted in everyday micro-interactions that can be depicted and then identified by students (Kayıran and Bağçeci, 2025; Emre et al., 2020).

The feasibility of pictorial approaches to values has historical precedent. The Picture-Based Value Survey for Children (PBVS-C) showed that visual materials can differentiate children’s value structures in developmentally appropriate ways (Döring et al., 2010). Yet most available pictorial tools focus on early childhood or global value preferences, not middle-school recognition of values embedded in social-school contexts. Meanwhile, adjacent literatures suggest that school climate is robustly linked to prosocial behavior and resilience during adolescence (Luo et al., 2023; Wang et al., 2022), implying that a recognition-focused pictorial assessment should relate to these constructs if it captures the intended domain.

Against this backdrop, the Pictorial Values Recognition Test (PVRT) is proposed to assess whether students in the middle-school years can correctly recognize values such as fairness, responsibility, respect, and helpfulness when presented with brief, realistic classroom and peer-interaction scenes. The development plan aligns with current standards for validity evidence (American Educational Research Association, American Psychological Association, and National Council on Measurement in Education, 2014; Sireci, 2023): (a) Test content and response processes will be established through expert review and cognitive interviews to ensure scenario clarity, cultural relevance, and unidimensional item intent; (b) Internal structure will be examined via EFA/CFA; (c) Reliability will be estimated through internal consistency and, where feasible, test–retest; (d) Relations to other variables will be tested against theoretically proximal constructs (school climate, empathy/prosocial behavior); and (e) Measurement invariance will be evaluated across sex and grade to support fair group comparisons. Transparent reporting of these steps answers increasing expectations in high-impact journals for comprehensive validity arguments and replicable psychometric evidence (Brown and Zhao, 2023; Sireci, 2023).

The theoretical rationale for pictorial recognition rests on emerging accounts of moral-value perception: perceivers use learned schemas to extract value-relevant information from social scenes, rapidly matching cues (e.g., equitable turn-taking, rule-keeping) to corresponding value labels (Young, 2021). Well-designed pictorial stimuli can therefore function as structured elicitors of these schemas. Moreover, the school context provides rich, culturally resonant situations queueing in the cafeteria, group-work roles, sharing materials where values are commonly expressed and observed, increasing ecological validity. If the PVRT captures these judgments reliably, it can serve educators as a formative tool and provide policy makers with metrics for values-education efforts, complementing broader school-climate or SEL evaluations (Kim and Park, 2024; Silke, 2024).

The 10 core values defined by the Ministry of National Education (MONE) justice, friendship, honesty, self-discipline, patience, respect, love, responsibility, patriotism, and helpfulness constitute the foundational elements of students’ socio-emotional and moral development. The 12 values assessed in this study were not derived solely from MONE’s framework; rather, they were selected through an integrated process that considered both national curriculum priorities and universal value theories, particularly Schwartz’s Theory of Basic Human Values. Following an extensive literature review and expert consultation, the values included in the PVRT were structured to align with developmentally meaningful expectations in the Turkish curriculum as well as well-established universal value dimensions such as universalism, benevolence, security, social order, self-regulation, and interpersonal harmony. Accordingly, values such as justice, responsibility, benevolence/helpfulness, patience, love, respect, friendship, honesty, self-discipline, patriotism, compassion, and tolerance represent a coherent framework that is both nationally grounded and theoretically global in scope. This alignment demonstrates that the PVRT reflects not only the priorities of the national curriculum but also a universally recognizable system for assessing value recognition.

Within Schwartz’s Theory of Basic Human Values, benevolence encompasses motivational tendencies such as helping others, showing concern for the welfare of close others, and acting with a sense of interpersonal responsibility. These characteristics parallel MONE’s conceptualization of helpfulness, indicating a strong conceptual overlap between national and universal frameworks. Similarly, Schwartz’s dimensions of universalism, security, conformity, and tradition correspond to several of the values assessed in the PVRT such as justice, patriotism, responsibility, respect, and self-discipline. Thus, the PVRT does not aim merely to assess MONE’s core values; rather, it evaluates a value recognition ability that is conceptually compatible with the motivational domains identified in international value research.

The selection of the 12 values included in the PVRT followed a three-stage process: (1) an examination of MONE’s framework of 10 core values; (2) an analysis of their conceptual correspondences within universal value theories, particularly Schwartz’s model; and (3) a developmentally informed evaluation of which values could be meaningfully represented through culturally appropriate visual scenarios for middle school students. The resulting set of values thus represents a developmentally appropriate, culturally sensitive, and theoretically grounded framework that bridges national educational priorities with universal value theory.

In sum, recent literature (2021–2025) converges on three points. First, values, school climate, and social–emotional competencies are reciprocally influential in early adolescence (Cheng, 2024; Luo et al., 2023; Oeschger et al., 2024; Wang et al., 2022; Kayıran, 2023). Second, visual/pictorial assessment can be both developmentally appropriate and psychometrically defensible when built with careful construct definition and modern validation practices (Lo and Huang, 2024; Simon et al., 2022). Third, journals increasingly expect integrated validity arguments spanning content, process, structure, and consequences (American Educational Research Association, American Psychological Association, and National Council on Measurement in Education, 2014; Brown and Zhao, 2023; Sireci, 2023). Addressing these points, the PVRT aims to fill a concrete gap by measuring values recognition through brief, school-realistic pictorial scenarios, thereby supporting classroom feedback loops and system-level program evaluation in values education.

The Pictorial Values Recognition Test (PVRT), developed within the scope of this study, conceptualizes value recognition not as an attitude or a personality trait but as a performance-based ability through which students correctly identify and match a value using visual cues. Accordingly, the PVRT is a Classical Test Theory (CTT)-based *recognition test* in which each item has a single correct answer. In such tests, construct validity is evaluated not through the identification of an underlying latent factor structure but through item-level indicators such as item difficulty, item discrimination, and internal consistency (KR-20). Therefore, the validity framework of the PVRT is grounded in content validity and CTT-based item statistics. This approach is widely accepted in the international literature on achievement and recognition testing.

The primary aim of this study is to present the development procedures, content validity evidence, and psychometric properties of the Pictorial Values Recognition Test (PVRT), which was designed to assess middle school students’ ability to recognize values through visual cues. The study contributes to the literature in two main ways: (1) It introduces an original measurement tool that evaluates values not through written attitude statements but through contextually and culturally adapted visual scenarios; and (2) it provides a developmentally appropriate, brief, practical, and visually based instrument that addresses the scarcity of assessment tools measuring value awareness at the middle-school level. In doing so, the PVRT enables a more experiential and context-sensitive evaluation of the outcomes of values education.

## Method

### Research design

This study employed a quantitative survey design to determine the value perceptions of middle school students. The survey model aims to describe the current status of individuals’ opinions, attitudes, knowledge, or skills within a given population or sample (Karasar, 1994).

### Study group

The study group consisted of a total of 336 middle school students enrolled in public schools located in the central districts of Kahramanmaraş during the 2025–2026 academic year. The demographic characteristics of the participants are presented below with corresponding percentages. In terms of gender distribution, the sample included 176 female students (52.38%) and 160 male students (47.62%). Regarding grade level, 85 students (25.30%) were in 5th grade, 90 students (26.79%) in 6th grade, 82 students (24.40%) in 7th grade, and 79 students (23.51%) in 8th grade. This distribution demonstrates that the study provides a balanced representation of the middle-school age group, with a meaningful number of participants from each grade level.

Given that the implementation of the study depended on school permissions, administrative procedures, and the researcher’s accessibility, the sample was selected using a convenience sampling method. This approach is commonly employed in school-based research and allows data collection from volunteer students in classrooms to which the researcher has access. Although convenience sampling does not provide full random representativeness, it is widely used in instrument development studies, particularly when the primary aim is to conduct validity and reliability analyses.

### Development of the data collection instrument: pictorial values recognition test

In this study, data were collected using a measurement tool developed by the researcher the Pictorial Values Recognition Test (PVRT). The development process of the test was carried out through the following systematic steps:

#### Determining the scope and selection of values

In the study, each visual developed for the 12 targeted values was created with direct expert involvement. Two visual arts specialists played an active role in translating the values into concrete pictorial representations. The experts contributed by designing the composition of each scene representing a specific value, ensuring the cultural appropriateness of character and setting designs, maintaining visual clarity and simplicity appropriate for the students’ developmental level, and depicting the value-related behavior in the most explicit and comprehensible manner. The visuals were produced through a three-stage process: Drafting, revision, and finalization.

In this study, 12 values were included in the development of the PVRT. Although the Ministry of National Education (MONE) identifies 10 foundational values within its values education framework, the present study expanded this set to 12 values. This decision was informed by a comprehensive review of national and international literature, evaluations of widely used value models particularly Schwartz’s Theory of Basic Human Values and expert judgments regarding developmental appropriateness and visual representability. Notably, the value of “helpfulness” corresponds conceptually to the “benevolence” category in Schwartz’s framework, illustrating how national curriculum-based values align with universal motivational domains. The resulting set of 12 values such as justice, respect, love, responsibility, benevolence/helpfulness, friendship, honesty, patience, self-discipline, patriotism, compassion, and tolerance provides a conceptually coherent and culturally sensitive framework that reflects both MONE’s educational priorities and the broader structure of universal value theory. Accordingly, the PVRT represents not merely a curriculum-based assessment tool but an instrument grounded in a theoretically robust and internationally recognizable value framework.

#### Expert review and content validity

To ensure content validity, an expert panel consisting of eight specialists was convened. The panel included three academics in the field of values education, two experts in measurement and evaluation, and two specialists in visual arts. The experts evaluated each visual in terms of its adequacy in representing the intended value, cultural appropriateness, developmental suitability for the target age group, and overall visual clarity. In addition, the measurement and evaluation experts examined whether each visual represented a single, unidimensional value and assessed the presence of any visual elements that might create misinterpretation or construct-irrelevant variance. Content validity was calculated using Lawshe (1975) method, based on Content Validity Ratio (CVR) and Content Validity Index (CVI) values; visuals with CVR values above 0.80 were retained. This process strengthened the content validity of the test and demonstrated that the visuals clearly represented their intended values.

#### Pilot application

- A pilot study was conducted with 50 middle school students to assess clarity, adequacy of completion time, and visual comprehensibility.
- Two illustrations that were reported as unclear were subsequently revised.

#### Scoring procedure

- Each student was provided with a test form containing 12 pictorial items.
- Students were asked to write which value each illustration represented.
- Each correct match was scored 1 point, while incorrect or blank responses received 0 points.
- The total possible score ranged from 0 to 12, with higher scores indicating a higher level of value recognition.

#### Reliability study

- Since the test consisted of dichotomously scored (right/wrong) items, the KR-20 reliability coefficient was calculated.
- Tests with KR-20 values above 0.70 were considered reliable (Büyüköztürk, 2002).
- Since the PVRT is a recognition-based performance test, each item includes a single correct answer and functions as a discrete performance indicator. For this reason, conducting factor analysis is not appropriate for establishing structural validity. Instead of examining latent constructs, the study employed Classical Test Theory (CTT) indices item difficulty (p), item discrimination (r), and the KR-20 internal consistency coefficient to evaluate the test’s psychometric adequacy. This approach parallels the measurement models used in achievement-based assessments such as PISA, TIMSS, and similar large-scale performance tests. The obtained KR-20 coefficient (0.87) indicates that the items measure a sufficiently homogeneous construct and that the instrument demonstrates strong internal consistency.

#### Construct validity

- Item–item correlation analyses were conducted, and items with low discrimination (r < 0.20) were revised or removed.

### Instrument development process

The Pictorial Values Recognition Test (PVRT) was developed to assess middle school students’ perceptions of values. The test comprised 12 visual items, each representing a different value (e.g., patriotism, respect, patience, etc.). The illustrations were selected and refined in consultation with three experts in values education and two experts in visual design, ensuring content validity and cultural relevance.

**Figure 1:**
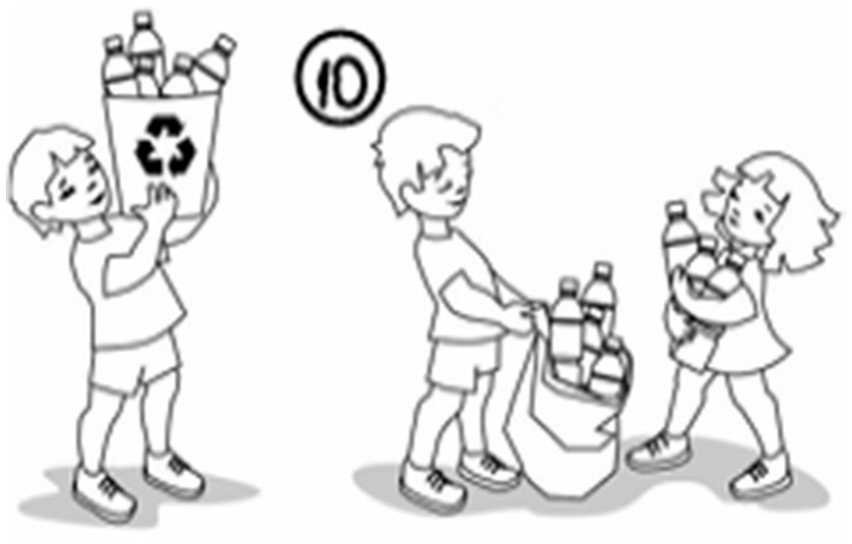
Sample visual for the value of responsibility (environmental awareness).

To demonstrate the representational strength and contextual clarity of the pictorial items used in the study, selected sample visuals from the PVRT are presented in Appendix 1. These examples illustrate how the values were depicted through everyday life scenarios and how each item was structured during the development process. All visuals were anonymized in accordance with ethical requirements and are shared solely for illustrative purposes. For instance, Figure 1 demonstrates how the value of *responsibility (environmental awareness)* was visualized through a contextual scenario. The visuals were designed to reflect social situations that middle school students can easily relate to in their daily lives, thereby enhancing the ecological validity and interpretability of the items.

In this visual, two children are shown collecting plastic bottles and placing them into a recycling bin. This scene contextually represents the values of *responsibility* (*environmental awareness*).

During data collection, the 12 illustrations were presented to students in a randomized order. Students were instructed to indicate which value each picture represented. A correct identification received 1 point, and an incorrect identification received 0 points. Consequently, total test scores ranged from 0 to 12, with higher scores reflecting a greater ability to recognize values through visual representations.

The development of the instrument followed standardized test construction procedures as outlined in the measurement and evaluation literature. These stages included:

- defining the scope and selecting values,
- creating a pool of items and illustrations,
- obtaining expert judgments and ensuring content validity,
- conducting a pilot study,
- analyzing item characteristics (KR-20 reliability, item difficulty, and discrimination indices),
- and performing validity–reliability studies before preparing the final test form.

**Figure 2:**
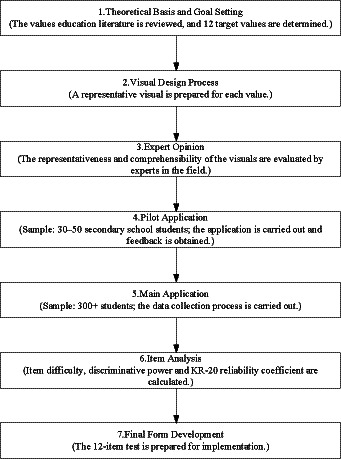
Diagram of the pictorial values recognition test (PVRT) development process.

The overall test development process is schematically illustrated in Figure 2.

This figure illustrates the sequential stages followed during the development of the Pictorial Values Recognition Test (PVRT). The process began with defining the purpose and scope, followed by creating a pool of visual items, obtaining expert feedback to ensure content validity, conducting the pilot study, and performing item analyses (KR-20 reliability coefficient, item difficulty, and discrimination indices). In the final stage, validity and reliability analyses were completed, and the finalized test form was prepared.

### Data collection instrument

The data collection instrument developed for this study was the Pictorial Values Recognition Test (PVRT). The test includes 12 original illustrations, each corresponding to one of the 12 values identified through an integrative process that combined the Turkish Ministry of National Education (2022) values education framework with evidence from national and international value theories and most notably Schwartz’s Theory of Basic Human Values. These values are justice, generosity, friendship, honesty, compassion, self-control, patience, respect, love, responsibility, patriotism, and helpfulness (see Appendix 1).

Students were asked to examine each illustration carefully and write down the name of the value they believed it represented. The scoring system was as follows:

- 1 point for a correct match between the picture and the corresponding value,
- 0 points for an incorrect match or unanswered item.

Thus, each student could receive a total score ranging from 0 to 12, where higher scores indicate a higher level of value recognition.

In this study, only one pictorial item was developed for each value. Although it is possible to create multiple draft visuals for each value during test development, a single-item structure was adopted at the outset to represent each value visually. Consequently, each item evaluates the targeted value through one visual scenario. This approach was preferred to keep the overall test length manageable and to reduce administration time; however, it also introduces certain limitations, particularly the inability to capture alternative visual representations or subdimensions of the same value.

### Validity and reliability studies

- Content validity was established through expert evaluation. Opinions were obtained from five academicians specializing in values education, measurement and evaluation, and visual arts, along with three middle school Turkish language teachers. The experts reviewed the alignment between each illustration and the value it was designed to represent.
- A pilot study was conducted with 50 students to assess clarity, suitability of duration, and comprehensibility.
- Reliability analysis was carried out using the Kuder–Richardson 20 (KR-20) coefficient to assess internal consistency.
- Item difficulty (p) and item discrimination (r) indices were calculated, and items with discrimination values below 0.20 were removed from the test.

### Scoring system and level classification

The PVRT was designed to measure middle school students’ ability to recognize values. Each of the 12 items represents one specific value: justice, generosity, friendship, honesty, compassion, self-control, patience, respect, love, responsibility, patriotism, and helpfulness.

Students were asked to identify the value depicted in each illustration. The scoring procedure was implemented as follows:

- Correct match: 1 point (each student’s total score = number of correct matches × 1)
- Incorrect match or blank response: 0 points
- Total possible score: ranges from 0 to 12

This scoring approach allows for a quantitative assessment of students’ value recognition level. The total score obtained from the scale is used to determine each student’s overall level of value perception.

In the field of values education, interpreting test scores through categorized performance levels is essential for meaningful evaluation (Dılmaç et al., 2007; Kuşdil and Kağitçibaşi, 2000).

Accordingly, students’ total scores were classified into three interpretive levels based on their recognition performance:

- Low level: 0–4 points
- Moderate level: 5–8 points
- High level: 9–12 points

This classification provides a clear framework for interpreting the degree to which students can accurately identify and associate values through visual representations.

This classification allows teachers to identify students’ current status regarding value recognition and to adapt their instructional plans accordingly. Specifically, students with low scores are considered to require additional support in values education. For those who scored at a moderate level, reinforcement activities are recommended in the value domains where they demonstrate weaknesses, while high-scoring students are encouraged to further develop their awareness through community service and social responsibility projects (Yeşil and Aydın, 2007).

### Data analysis

The data obtained from the PVRT were analyzed using the SPSS 25.0 statistical software package. First, descriptive statistics (frequency, percentage, arithmetic mean, and standard deviation) were calculated. To compare mean scores across demographic variables such as gender and grade level, independent samples t-tests and one-way analysis of variance (ANOVA) were performed. The significance level for all statistical tests was set at *p* < 0.05.

## Findings

### Descriptive statistics

Table 1 presents the descriptive statistics for the 12 values assessed through the *Pictorial Values Recognition Test (PVRT)*. For each value, the minimum, maximum, mean, standard deviation, skewness, and kurtosis values are reported. The analysis was conducted on data collected from 336 middle school students.

**Table 1:** Pictorial values recognition test: scoring and level classification.

Score range	Level description	Explanation
0–4	Low Level	The rate of value recognition is low; students show insufficient awareness and understanding of fundamental value concepts.
5–8	Moderate Level	The rate of value recognition is moderate; students recognize some values, but certain conceptual gaps and misunderstandings remain.
9–12	High Level	The rate of value recognition is high; students demonstrate a well-developed ability to accurately identify and understand fundamental value concepts

The score ranges were determined based on the total number of correct responses obtained from the test.

The skewness and kurtosis coefficients for all items remained within the acceptable range of ±2, indicating that the dataset approximates a normal distribution (George and Mallery, 2010). This supports the suitability of the data for subsequent parametric statistical analyses.

According to the analysis results, the value with the highest mean score among students was patriotism ( $\bar{X}$ = 0.93), whereas the value with the lowest mean score was helpfulness ( $\bar{X}$ = 0.57). The skewness and kurtosis coefficients for all items were within the acceptable range of ±2, indicating that the data were approximately normally distributed (George and Mallery, 2010).

A noteworthy finding of the study is that students’ recognition level for the value of *helpfulness* was considerably lower than for the other values ( $\bar{X}$ = 0.57). This outcome suggests that the relatively reduced recognition of a culturally salient value may be associated with the clarity or contextual explicitness of the visual stimulus used to represent it. The finding raises the possibility that the pictorial scenario for helpfulness may not have conveyed the intended behavioral cue with sufficient distinctiveness, thereby affecting students’ ability to correctly identify the value.

This finding suggests that the item distribution within the test was balanced, and that the instrument consistently measured students’ ability to distinguish among different values.

Table 2 presents the distribution of students’ value recognition levels based on their total scores from the *Pictorial Values Recognition Test (PVRT)*. According to the scoring system, total scores were classified into three categories:

- Low level: 0–4 points
- Moderate level: 5–8 points
- High level: 9–12 points

**Table 2:** Descriptive statistics for the pictorial values recognition test (*n* = 336).

Values	N	Min	Max	Mean	SD	Skewness		Kurtosis	
						Statistic	S. E	Statistic	S. E
Justice(1)	336	0	1	0.71	0.46	−0.91	0.13	−1.19	0.27
Generosity(2)	336	0	1	0.60	0.49	−0.42	0.13	−1.84	0.27
Friendship(3)	336	0	1	0.65	0.48	−0.63	0.13	−1.62	0.27
Honesty(4)	336	0	1	0.63	0.48	−0.55	0.13	−1.71	0.27
Compassion(5)	336	0	1	0.63	0.48	−0.55	0.13	−1.71	0.27
Self-Control(6)	336	0	1	0.60	0.49	−0.39	0.13	−1.86	0.27
Patience(7)	336	0	1	0.59	0.49	−0.35	0.13	−1.89	0.27
Respect(8)	336	0	1	0.63	0.48	−0.55	0.13	−1.71	0.27
Love(9)	336	0	1	0.52	0.50	−0.10	0.13	−2.00	0.27
Responsibility(10)	336	0	1	0.67	0.47	−0.73	0.13	−1.48	0.27
Patriotism(11)	336	0	1	0.93	0.26	−1.26	0.13	1.67	0.27
Helpfulness(12)	336	0	1	0.57	0.50	−0.30	0.13	−1.92	0.27

SD, Standard deviation; SE, Standard error.

The analysis findings revealed that the majority of students (64.3%) demonstrated a moderate level of value recognition, while 32.7% were at a high level, and only 3% were at a low level.

This distribution indicates that the *Pictorial Values Recognition Test (PVRT)* was able to effectively differentiate students’ value recognition levels, and that, overall, participants exhibited a moderate-to-high awareness of values (Table 3).

**Table 3:** Students’ value recognition levels according to the pictorial values recognition test (*n* = 336).

Levels	n	%
Low Level	10	3.0
Moderate Level	216	64.3
High Level	110	32.7
Total	336	100.0

### Item analysis

Table 4 presents the results of the item analysis for the *Pictorial Values Recognition Test (PVRT)*. For each item, the item difficulty index, item discrimination index, and the KR-20 reliability coefficient of the entire test were calculated.

**Table 4:** Item analysis results of the pictorial values recognition test (*n* = 300).

Item no	Represented value	Item difficulty (p)	Item discrimination (r)	Decision
1	Justice	0.72	0.46	Accepted
2	Generosity	0.68	0.42	Accepted
3	Friendship	0.75	0.50	Accepted
4	Honesty	0.70	0.48	Accepted
5	Compassion	0.65	0.40	Accepted
6	Self-control	0.58	0.38	Accepted
7	Patience	0.60	0.41	Accepted
8	Respect	0.78	0.52	Accepted
9	Love	0.82	0.55	Accepted
10	Responsibility	0.66	0.44	Accepted
11	Patriotism	0.63	0.43	Accepted
12	Helpfulness	0.80	0.53	Accepted

The KR-20 coefficient represents the internal consistency of the entire test. The obtained value of KR-20 = 0.87 indicates that the Pictorial Values Recognition Test (PVRT) possesses a high level of internal reliability.

The KR-20 coefficient was found to be 0.87, indicating a high level of internal consistency (Büyüköztürk, 2002). The item difficulty indices ranged from 0.58 to 0.82, while the item discrimination indices varied between 0.40 and 0.55.

These results demonstrate that the items were generally of moderate difficulty and exhibited high discriminative power, confirming that the PVRT reliably measures students’ ability to recognize and differentiate among values.

The item difficulty indices ranged from 0.58 to 0.82, suggesting that the test items were of moderate difficulty overall. The item discrimination indices varied between 0.38 and 0.55, with all items meeting the acceptable criterion (> 0.30). These results demonstrate that the items were sufficiently capable of measuring students’ levels of value recognition. The high internal consistency (KR-20 = 0.87) further confirms that the PVRT is a reliable measurement instrument for assessing middle school students’ ability to recognize values.

## Discussion

The findings from the *Pictorial Values Recognition Test (PVRT)* administered to middle school students provide valuable insights into students’ ability to recognize and internalize fundamental values within educational contexts. The results showed that the majority of students demonstrated moderate (64.3%) and high (32.7%) levels of value recognition. This distribution suggests that while students possess a foundational ability to identify various value concepts, certain values such as justice and compassion may be less well understood compared to others. This outcome aligns with previous research emphasizing that values education must employ effective strategies to strengthen the understanding and application of values in real-life contexts (Mansyur et al., 2021; Suyatno et al., 2019).

The comparatively lower recognition rates observed for abstract values such as *justice* and *compassion* are consistent with well-established findings in developmental psychology. Middle school students (ages 11–14) are in the transitional period toward Piaget’s *formal operational stage*, during which the ability to reason systematically about abstract moral principles becomes possible but is not yet fully consolidated (Piaget, 1972). As a result, students tend to more readily identify visual stimuli depicting concrete, observable behaviors, whereas they may struggle to derive the intended meaning from contextual cues that require abstraction, perspective-taking, or inferential reasoning. This developmental pattern is further supported by Kohlberg (1984) theory of moral development, which posits that individuals in this age group predominantly operate at the *conventional level*, where moral judgements rely on social norms, rule adherence, and authority approval rather than internally constructed moral principles. Consequently, pictorial scenarios intended to represent complex constructs such as distributive fairness for justice or affectively nuanced care for compassion may be interpreted inconsistently by students, reflecting normative developmental variability rather than a psychometric limitation of the PVRT. These findings highlight the importance of aligning visual value assessments with students’ cognitive and moral developmental profiles and underscore the need for continued refinement of visual stimuli representing abstract moral principles.

The 12 values included in the PVRT were not derived solely from the Ministry of National Education’s (MONE) framework of 10 core values; rather, they were identified through an integrative process that involved examining national curriculum priorities alongside international value theories. In Schwartz’s Theory of Basic Human Values (1992), values such as *helpfulness*, *responsibility*, and *compassion* align closely with the motivational domains of benevolence and universalism, both of which emphasize prosocial concern and the protection of others’ welfare. Similarly, values such as *justice* and *respect* correspond to the universalism and conservation–social order dimensions, which include fairness, rule-based coordination, and interpersonal respect. *Patriotism*, while culturally contextual, aligns conceptually with Schwartz’s security, ingroup belonging, and collective identity value clusters (Schwartz et al., 2012). This broader theoretical grounding demonstrates that although the PVRT reflects national educational priorities, it simultaneously embodies value constructs that are recognizable and theoretically coherent within the universal value literature. Thus, the PVRT offers a dual-layered measurement structure both culturally situated and conceptually universal which enhances its interpretive strength and cross-cultural relevance.

The findings related to students’ recognition of the value of *helpfulness* can also be understood within the framework of Schwartz’s benevolence category, which encompasses motivations such as helping others, acting cooperatively, and promoting the welfare of close others (Schwartz, 1992; Schwartz et al., 2012). In this sense, the visual representation of helpfulness used in the PVRT corresponds not only to the national curriculum’s conceptualization of the value but also to its broader meaning in the universal value literature. This alignment underscores the conceptual robustness of the PVRT indicators and supports the theoretical grounding of the study’s interpretation of the helpfulness-related findings.

The relatively lower recognition scores for abstract values such as *justice* and *compassion* may partially reflect the inherent challenges associated with visually representing abstract moral constructs. Unlike concrete prosocial behaviors, abstract values often involve complex social judgments, relational subtleties, and inferred intentions elements that are difficult to fully capture within a single pictorial frame. These interpretive demands can result in greater variability in student responses, particularly in single-item assessments. Developmental research supports this interpretation: adolescents’ ability to reason about abstract moral principles develops gradually (Kohlberg, 1984; Wainryb and Turiel, 1994), and abstract concepts are less consistently recognized than concrete behaviors during early adolescence. For future work, the creation of visual item pools containing multiple exemplars for each abstract value followed by systematic piloting and selection of the strongest items would improve representational clarity and enhance the content validity of visual assessments targeting abstract moral constructs.

Verification of the test’s reliability through the KR-20 coefficient and item analysis underscores the potential of visual-based instruments in educational assessment. Such tools can make abstract concepts often challenging for students more accessible and cognitively meaningful. Integrating visual materials into pedagogical strategies not only aids recognition but also fosters deeper engagement with the content, consistent with the growing body of literature advocating integrated values education across subjects (Yaman and Anilan, 2021; Brady et al., 2011). The conscious use of interactive and visual pedagogical tools is crucial in enhancing students’ moral reasoning and their ability to internalize values (Thornberg and Oğuz, 2016).

Given the structural characteristics of the PVRT, each item functions as a performance-based recognition task with a single correct answer. Therefore, applying factor analysis which assumes an underlying latent construct measured by multiple continuous indicators is not appropriate for this type of instrument. Instead, validity was established through content validity procedures and Classical Test Theory (CTT) indices, including item difficulty, item discrimination, and internal consistency. This approach aligns with international measurement standards for recognition and achievement tests, where construct validity is evaluated through content representativeness and item-level psychometric performance rather than latent factor modeling.

Furthermore, the effectiveness of values education is significantly influenced by the sensitivity of the learning environment and teachers’ perspectives. Studies have shown that educators who explicitly model values in their instructional practices contribute markedly to students’ understanding and adoption of those values (Görgüt and Tutkun, 2018; Saputri and Marzuki, 2021). This reinforces the need for teacher education programs and curricula that prioritize values education across disciplines, moving beyond traditional moral or social studies courses (Solissa, 2022).

Evidence from various cultural contexts also supports the idea that systematic value clarification initiatives are integral to holistic human development. Across educational paradigms, a shared goal emerges: to nurture character formation and prepare students as responsible, ethically grounded citizens. Accordingly, developing a visual-based recognition framework serves as both a theoretical and practical approach to fostering students’ sense of social responsibility and community engagement (Schuelka and Sherab, 2022; Siswati et al., 2022).

For educational policymakers and practitioners, these findings highlight the necessity of designing instructional strategies that combine comprehensive discussions on values with visual stimuli and contextual learning. This approach ensures that students not only recognize but also deeply internalize essential moral and civic principles. The integration of visual tools with abstract moral instruction presents a promising pathway for cultivating ethically conscious and socially responsive individuals capable of navigating complex moral landscapes with clarity (Paleeri, 2017).

In conclusion, the present study reinforces the growing role of visual-based recognition tools in values education and advocates for the continuous refinement of instructional methodologies. Such an emphasis can ultimately enhance students’ understanding of values and contribute to the development of well-rounded individuals prepared for the dynamic demands of modern society.

The use of a single pictorial item to represent each value in the PVRT offers practical advantages in terms of administration time and cognitive load; however, it also introduces certain psychometric limitations. Students may conceptually understand a value yet interpret the behavior depicted in a single visual scenario in different ways, leading to variability unrelated to the construct being measured. For this reason, future research should consider developing a larger item pool with multiple visual representations for each value, followed by systematic piloting and item refinement. Such an approach would enhance representational breadth and improve the instrument’s overall construct coverage.

An examination of recognition rates revealed that *patriotism* yielded one of the highest levels of correct identification, whereas *helpfulness* was recognized at a markedly lower rate compared to other values. Given the prominent role of helpfulness in Turkish cultural norms related to social cohesion and communal support, this finding is particularly noteworthy. The lower recognition of this value may be linked to the visual stimulus itself, suggesting that the depicted behavior may not have been sufficiently clear or distinctive for students. Misinterpretation is especially likely in single-item assessments where alternative behavioral cues cannot be triangulated across multiple scenarios. Therefore, refining the visual representation of helpfulness by designing a more explicit, contextually transparent, and unambiguously prosocial scenario is recommended for future iterations of the test.

The findings of the present study indicate that the PVRT has preliminary utility for assessing middle school students’ ability to recognize specific values through visual cues. However, the results should not be interpreted as evidence that the PVRT captures the entirety of students’ value development or fully represents all domains of value education. The findings reflect only the visual items used in this study and the characteristics of the specific sample. Factors such as the single-item structure, the variability in visual representations, and the later cognitive maturation of abstract moral concepts must be taken into consideration when interpreting the results. Accordingly, the PVRT should be regarded as an initial indicator of value recognition rather than a comprehensive measure. Future research incorporating multi-item visual pools, diverse samples, and iterative psychometric validation procedures would provide a stronger evidence base and further enhance the robustness of the instrument.

## Conclusion and recommendations

This study aimed to develop and implement the *Pictorial Values Recognition Test (PVRT)* to assess middle school students’ ability to recognize 12 values: justice, generosity, friendship, honesty, compassion, self-control, patience, respect, love, responsibility, patriotism, and helpfulness. Total scores on the test ranged from 0 to 12, with levels classified as low (0–4), moderate (5–8), and high (9–12).

The results revealed that most students (64.3%) demonstrated a moderate level of value recognition, 32.7% showed a high level, and only 3% performed at a low level. These findings indicate that while students can partially identify value concepts correctly, certain values may still be conceptually confusing. The KR-20 reliability coefficient, along with item difficulty and discrimination analyses, confirmed that the PVRT is a reliable and valid measurement instrument.

The validity of the PVRT was supported through Classical Test Theory–based item analyses and comprehensive content validity procedures. Unlike latent personality or attitudinal scales, where construct validity is typically established through factor-analytic methods, the structural validity of the PVRT was evaluated in terms of the degree to which each item consistently and accurately represents the targeted value. In recognition-based performance tests with single-correct-response items, validity is derived not from latent factor structures but from the clarity, representational accuracy, and psychometric performance of the individual items.

The findings also support the use of visual-based assessment tools as an effective means of concretizing abstract concepts such as values. Accordingly, the role of visual materials in value recognition and internalization processes is becoming increasingly important in educational contexts.

### Recommendations

1. For Curriculum and Instruction
  - It is recommended that visual materials be systematically integrated into values education activities at the middle school level.
  - Additional class activities should focus on values that were less accurately recognized by students to reinforce understanding.
2. For Measurement and Evaluation
  - The PVRT can serve as a practical tool for identifying students’ levels of value recognition and can be adapted for different age groups.
  - Similar visual-based tests can be developed to assess other sets of values across diverse educational contexts.
3. For Researchers
  - Conducting studies in different regions and socioeconomic settings can help reveal cultural variations in students’ value perceptions.
  - Longitudinal research is recommended to track changes in students’ value recognition over time.
4. For Policy and Curriculum Developers
  - Incorporating visual-based assessment tools into the national values education curriculum could support the joint development of students’ cognitive and affective domains, thereby fostering more comprehensive character formation (Yeşil and Aydın, 2007).

### Limitations

One of the primary limitations of this study is that each value was assessed using a single pictorial item. Given that concepts such as justice, helpfulness, or responsibility are inherently multidimensional, students may conceptually understand a value yet interpret the behavior depicted in the visual scenario in different ways. The absence of a multi-item structure restricts the opportunity to represent each value across diverse contexts and behavioral expressions. Consequently, measuring a value through a single visual exemplar may not fully capture the breadth and nuance of students’ value recognition. Future research should prioritize developing multiple pictorial items for each value and selecting the strongest-performing items through iterative piloting and item analysis to enhance the psychometric robustness of the instrument.

Another limitation concerns the relatively low recognition level observed for the value of *helpfulness*. This finding suggests that the visual stimulus representing helpfulness may have been interpreted differently by students. Because only one visual was used to depict this value, the scenario may not have sufficiently reflected the range of behaviors through which helpfulness is typically expressed. This issue is directly linked to the single-item design and highlights the need for more varied visual representations, particularly for culturally salient values. Expanding the item pool to include multiple contextual depictions of helpfulness would allow for more accurate measurement and reduce the risk of misinterpretation.

## Figures and Tables

**Appendix FIGURE 1:**
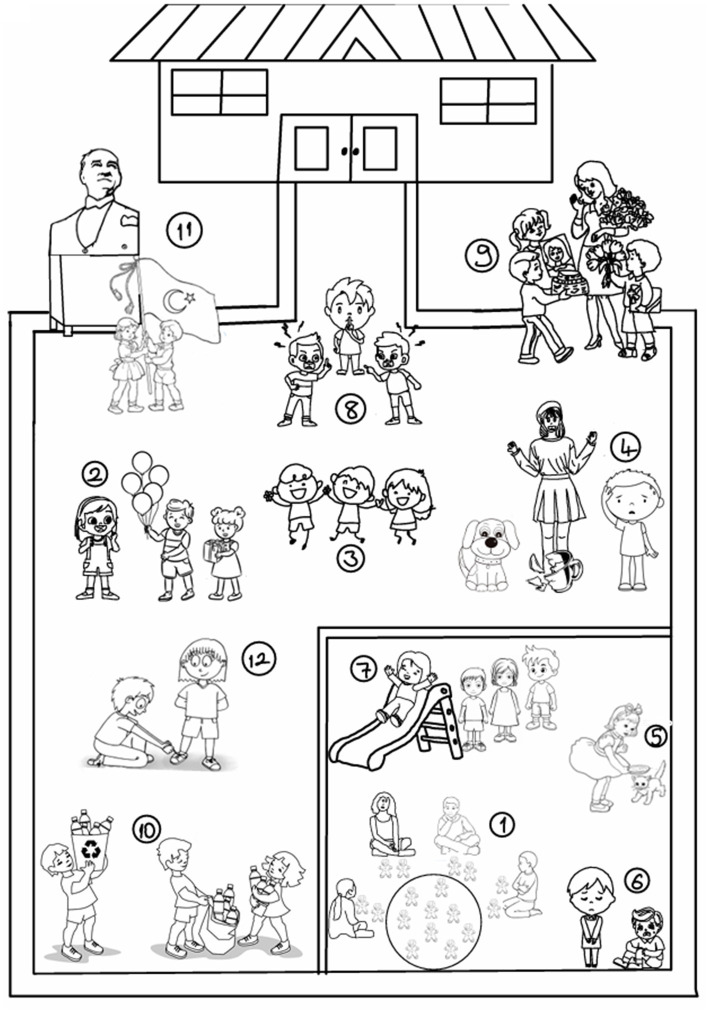
Pictorial items.

## Data Availability

The raw data supporting the conclusions of this article will be made available by the authors, without undue reservation.
